# Influence of Anticorrosive Pigment, Dry-Film Thickness and Conditioning Time on Protective Properties of Two-Component Epoxy Primer

**DOI:** 10.3390/ma15093041

**Published:** 2022-04-22

**Authors:** Tomislav Šolić, Dejan Marić, Ivan Peko, Ivan Samardžić

**Affiliations:** 1 Mechanical Engineering Faculty in Slavonski Brod, University of Slavonski Brod, Trg I. B. Mažuranić 2, 35000 Slavonski Brod, Croatia; dmaric@unisb.hr (D.M.); isamardzic@unisb.hr (I.S.); 2 Faculty of Science, University of Split, Ruđera Boškovića 33, 21000 Split, Croatia; ipeko@fesb.hr

**Keywords:** two-component epoxy coating, anticorrosive pigment, dry-film thickness, conditioning time, assessment of coating deterioration

## Abstract

The main protective properties of two-component epoxy coating are connected by the formation of a barrier of a certain thickness between the material and aggressive, environmentally induced reactants. Anticorrosive pigment is added to the coating in order to improve its protective effects. The conditioning time refers to the time interval required for the achievement of satisfactory cohesion bonds between the coating components, as well as a satisfactory adhesion force between the coating and the base material surface. This paper presents insights obtained after experimental research into the influence of input variables (the content of anticorrosive pigment in the coating, dry-film thickness, and conditioning time) on corrosion resistance. The specimens were kept in the aggressive atmosphere of a salt-spray test chamber within time intervals of 120, 240 and 480 h, where they were cyclically sprayed with a 5% sodium chloride (NaCl) solution, and then examined in laboratory conditions. Such a procedure imitated the aggressive conditions of a service environment. After exposure in the salt-spray test chamber, the specimens were tested to determine the protective properties of the coating and to evaluate damage occurring on the coating, with the purpose of assessing the coating quality in relation to the stated input variables. At all times, when the test samples were exposed to the salt chamber atmosphere, the anti-corrosion pigment content was found to have the greatest influence with the thickness of the dry coating film. The conditioning time was an influential factor to a lesser extent, and only in some observed cases. By analyzing the interactions of the input variables and the results obtained based on mathematical models and reaction surfaces, it was possible to define the most optimal values of the input parameters. For example, after 480 h of exposure in a salt chamber, notch corrosion of 0.6 mm was observed at a dry-film thickness of D1 and an anti-corrosion pigment content of 10%.

## 1. Introduction

Corrosion is a major problem occurring in all steel structures, as well as in all phases of steel production and service, from steel storage to the service lifecycle of any steel product [1,2]. Some studies have determined that the costs associated with corrosion amount to USD 2505.3 billion, which corresponds to 3.4% of the global GDP. These studies also conclude that appropriate methods and technologies applied to prevent the corrosion mechanisms could save 15–35% of total annual costs on a global scale, i.e., the corrosion-related costs could be reduced by USD 375–875 billion. The study on corrosion-related costs was a sort of follow-up of studies conducted in India, the United States, Japan, Kuwait, and the United Kingdom, which presented data on damage caused by corrosion in the agricultural, industrial, and service sectors [3,4]. Those studies not only proved the burden of corrosion on financial resources on a global scale, but also emphasized the need to improve procedures and technologies for corrosion prevention. For example, if a steel structure is in contact with seawater, corrosion mechanisms will be significantly accelerated because of the increased conductivity caused by dissolved chloride, which facilitates fast corrosion penetration into the material. Increased conductivity is favorable for the development of the destructive electrochemical mechanisms of corrosion, which result in the deterioration of material and deviations from its demanded and dimensional properties [5,6]. In order to avoid this, it is necessary to apply technologies for surface protection that will increase materials’ resistance to corrosion. Organic coatings are a method of surface protection that are applied for the protection of 3/4 of all metal surfaces [7]. This technology is also applied in industries such as the automotive industry, aeronautics, shipbuilding, the oil industry, etc. [8,9,10,11]. Epoxy coatings are given the greatest importance among organic coatings, because they have good mechanical properties, high thermal stability, good chemical and corrosion resistance, and are relatively cheap in terms of production costs [12,13]. Previous research has obtained good results in the application of two-component epoxy coatings in different atmospheric conditions, as such coatings facilitated better corrosion resistance and preservation of steel in coastal, industrial, and urban atmospheres [14,15]. The fact that epoxy coatings are very common in various applications is also shown by the fact that constant work is being conducted to improve the characteristics of epoxy binders [16]. The individual effect of other components such as anticorrosive pigments is also studied, with the aim of improving the adhesion properties [17]. Improving the adhesive properties can be linked to the protective effect of the coating, as it is known that the base coat must have good adhesion in order to be stable on the material, and thus, ensure its protective role. To achieve better adhesion in order to improve the protective properties, some authors have studied the use of cross-cut [16,18], and others pull-off methods [19,20]. In addition to varying the characteristics, i.e., the chemical composition of the studied coatings, authors have tried to achieve adhesive and, later, protective properties, with adequate methods and parameters of surface treatment to which the coating is applied [19,21]. All this research points to the fact that importance is attached to testing and improving the protective properties of epoxy primers.

Pigments, as one of the components of coatings, increase the protective properties of coatings [17]. The primarily purpose of coatings is to form a barrier to protect the base material from contact with aggressive reactants from the environment. Damage to the coating, either caused by mechanical actions or by exposure to an aggressive environment, would expose the base material to corrosion mechanisms again. In order to prevent this, pigments with anticorrosive action are added to the coating, thus helping to form a protective layer between the base material and the coating itself. The first pigments added to coatings were toxic, so it was necessary to replace them. Accordingly, references are made to studies that investigate the quality of replacing toxic zinc chromates with non-toxic zinc phosphates [22,23,24,25], which are the most-represented in this group [26]. There have been many tests performed on zinc phosphates in order to achieve their best anticorrosive effect and protective properties. One example of their usage is in coatings applied to welded joints in pipelines. The addition of 6% pigments to a coating achieved a stable phosphate film on the metal surface which, consequently, increased resistance to the transfer of electrolytes 5 to 10 times [27]. There are also other benefits to pigments studied. Tests have proved that, already, the addition of 2.6% pigment along with appropriate additives [28], or the addition of 3.5% of pigment in aqueous coatings [29], improved the protective properties of coatings. Some authors have increased the content of pigment in coatings, which led to better protective properties of the coating being achieved with the addition of 5% anticorrosive pigment [30]. Other studies have focused on the analysis of the effects reached by combining submicron-sheet zinc phosphate pigments and conventional zinc phosphate pigments at contents of 5, 10, 20 and 30%, within which the best results were obtained for coatings that contained 10% anticorrosive pigment [31]. Another test was also performed to confirm the obtained quality of the protective properties of coatings with the addition of 10% pigment [32].

The prevention of harmful corrosion mechanisms on steel zinc phosphates can be achieved through several different mechanisms [33]:

- Phosphate ion donation—This protective mechanism can only be used with ferrous metals. As water passes through the coating, there is a slight hydrolysis of zinc phosphate, resulting in secondary phosphate ions. These phosphate ions then form a protective passive layer which, when dense enough, prevents anodic corrosion. The porosity of phosphate coatings is closely related to the protective properties of the coating. The approximate formula of the phosphated metal compound is Zn_5_Fe(PO_4_)_2_∙4H_2_O;
- Formation of protective films on the anode—This mechanism is characterized by the adsorption of dissolved oxygen in the film on the metal surface. A heterogeneous reaction occurs to form a protective film of γ-Fe_2_O_3_; this thickens until it reaches an equilibrium value of 20 nm, which is sufficient to prevent external diffusion of iron. Therefore, the conclusion is that phosphate ions here do not directly contribute to the formation of the oxide film, but act on its completion or maintenance by closing the discontinuity with anionic precipitates of Fe (III) ions;
- Inhibitory aqueous extracts formed with certain oleoresin binders—Binder components, such as carboxyl and hydroxyl groups, form complexes either with zinc phosphate or with intermediates formed when zinc phosphate becomes hydrated and dissociated. These complexes can then react with the corrosion products to form a firmly adhering inhibitory layer on the substrate; or
- Substrate polarization—A mechanism characterized by the formation of an almost insoluble base salt that adheres well to a metal surface. The resulting salts limit the access of dissolved oxygen to metal surfaces and polarize the cathode regions.

**Figure 1:**
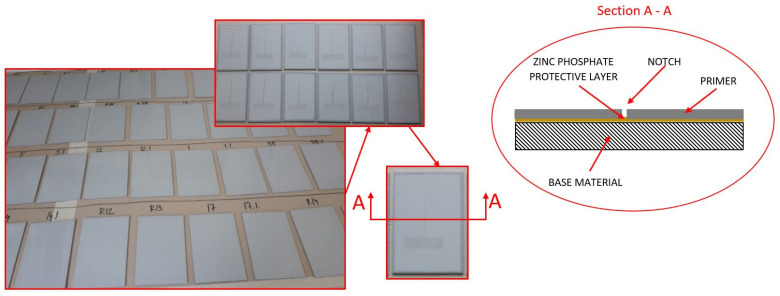
Protective mechanism of the primer with zinc phosphate anticorrosive pigment.

Figure 1 shows the protective mechanism of the primer with a zinc phosphate anticorrosive pigment.

Conditioning time refers to the period of time that is required after applying the coating on the base material, so that the coating can achieve satisfactory cohesion forces within the coating itself, as well as between the coating and the base material surface. If there is anticorrosive pigment contained in the coating, the satisfactory conditioning time also represents the period required for the creation of quality chemical compounds at the layer between the base material and the coating, i.e., for the creation of phosphates that inhibit corrosion mechanisms. In order to achieve the best possible results, authors have tested various conditioning time values. They start with testing for 24 h at room temperature, adding 8 h at 60 ± 2 °C [34], or 24 h at 25 °C, with an additional 24 h at 60 °C [35]. Slightly higher conditioning times for the testing of coatings are 5 days [36], 7 days [32], and 7 days at room temperature with one additional day at 100 °C [37]. Significantly higher conditioning times are 14 days [38] and 15 days [39]. Since the authors in previous studies varied the conditioning time from 1 to 14 days, a time of 7 days, i.e., 168 h, was chosen as the mean value for the planning of this experiment. According to this value, the values of 72 and 264 h were chosen as the lower and higher levels of experiment planning. Furthermore, the values of 32 and 304 h were derived by planning a centrally composite design test plan (CCD), as shown in Table 1.

The choice of optimal dry-film thickness was also influenced by the components of the coating, so it was necessary to find the right ratio of thickness and content of certain components that will provide the best protective properties. The values tested in previously conducted studies ranged from 60 ± 6 µm [40] to 120 µm [41], 130 ± 5 µm [35], 170 ± 10 µm [30], and up to 190 µm [14]. In reviewing previous research, some authors examine the individual effects of some of the variables defined here. In contrast, in this experimental study, their effects were examined by interaction. In this way, it is possible to correctly define the values of the individual input variables in the first place, i.e., to compensate for the reduction in the content of anticorrosive pigments (e.g., the dry-film thickness or the conditioning time), which will provide satisfactory protective properties.

## 2. Experimental Research

Within this experiment, the obtained results will be analyzed to establish the functional dependence in the form of a mathematical model, which will further determine the dependence of tested property on the input variables. The response surface methodology (RSM) was applied as a method of experiment planning, i.e., the central composite design (CCD) was chosen. Table 1 provides an overview of the coded values of factors, while Table 2 shows all the experimental runs with an overview of the obtained research results. It is important to note that the proportion of anticorrosive pigment is expressed in mass fraction.

The first step in running the tests according to the defined experiment design was the preparation of the specimens. The base material used for the preparation of the specimens was a general construction steel S235JR, the chemical composition of which is shown in Table 3.

**Figure 2:**
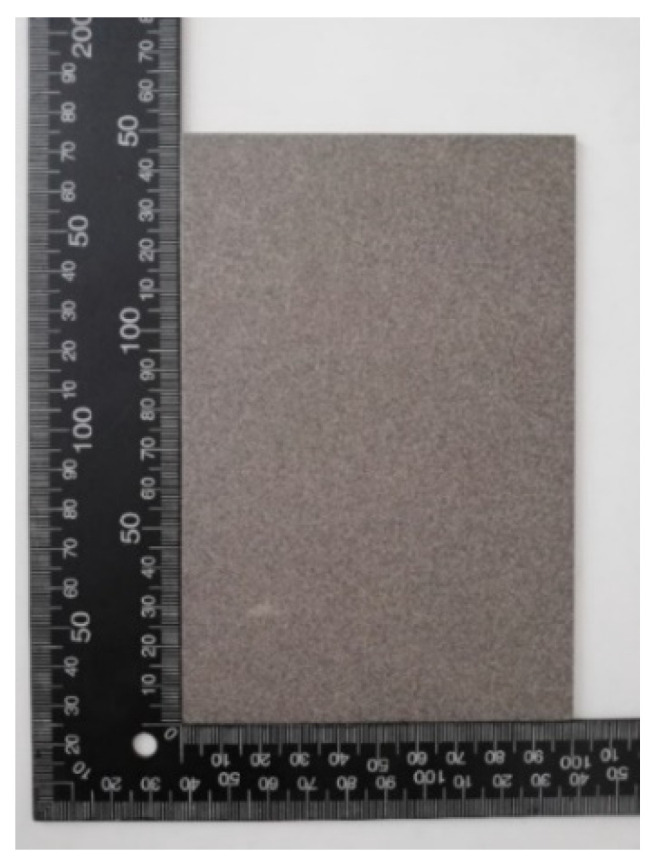
Test sample after abrasive jet treatment.

The specimens were cut to dimensions of 150 × 100 × 2 mm and mechanically treated with an abrasive jet, in order to achieve satisfactory quality of surface for the application of the protective coating. The surface was prepared according to the HRN EN ISO 12944-4 standard, with the required quality of Sa 2.5 according to the HRN ISO EN 8501-1 standard. As part of the visual inspection of the surface and comparison with the standard (etalon), the treated surface had a roughness in the range of 40–70 µm. The measuring of the roughness on each specimen was repeated 20 times, and then the mean value was calculated. Figure 2 shows the test sample after surface treatment with an abrasive jet.

**Figure 3:**
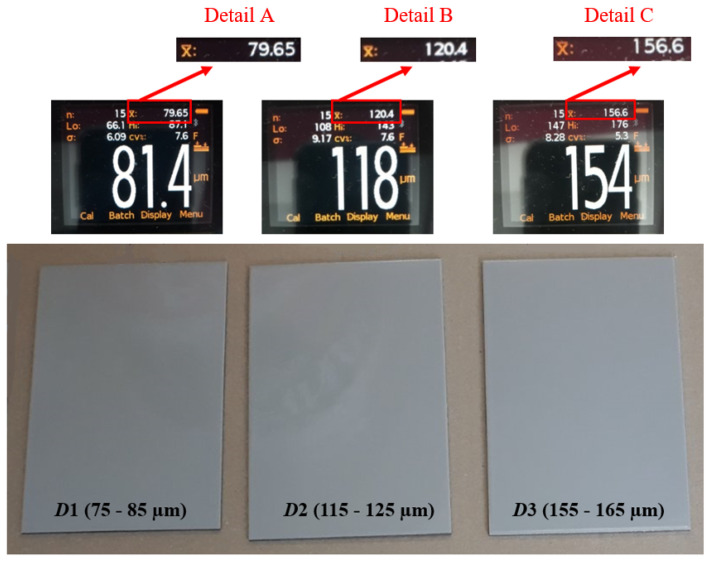
Test specimens after coating.

After inspecting the specimens’ surface quality, the next step was the application of the protective coating using airless spraying. The ratio of base and hardener was 5:1. As shown in Table 1, the dry-film thickness was varied at three levels: *D*1, *D*2, and *D*3. The dry-film thickness marked with *D*1 refers to specimens with a mean value ranging from 75 to 85 µm; *D*2 indicates specimens with a dry-film thickness mean value ranging from 115 to 125 µm; while *D*3 indicates specimens with a dry-film thickness mean value ranging from 155 to 165 µm. Figure 3 shows the test specimens after the application of the coating, with performed dry-film thickness measurements, to establish that everything is in accordance with the parameters defined by the test plan.

Each figure shows that 15 measurements were performed for the test sample, which is marked *n*; the minimum measured value is marked *L*o; the maximum measured value is marked *H*i; and the mean measured value is marked $\bar{x}$.

**Figure 4:**
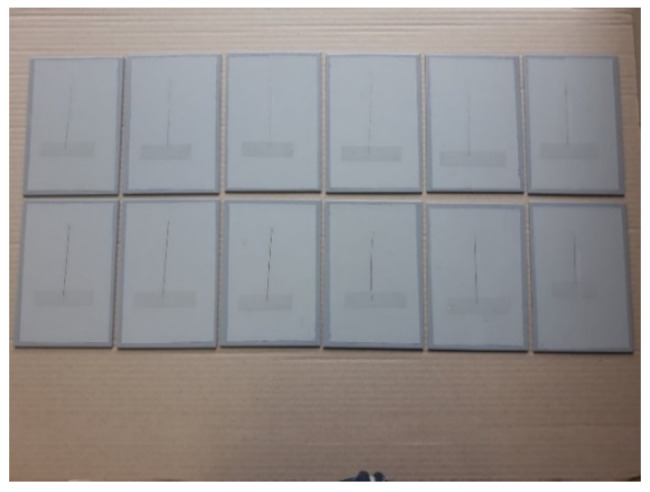
Prepared specimens before being placed in the salt-spray test chamber.

**Figure 5:**
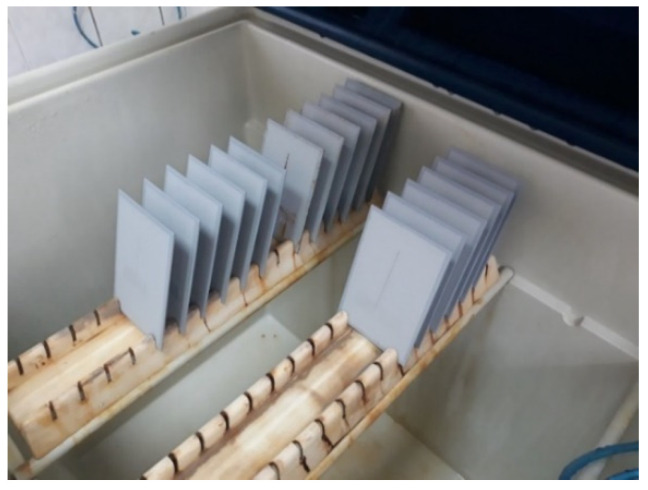
Test specimens inside the salt chamber workspace.

**Figure 6:**
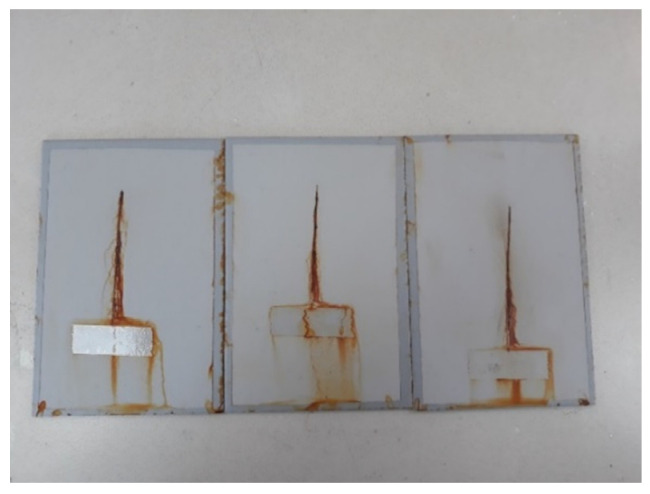
Specimens after exposure to the salt-spray test chamber.

This experiment focused on testing the coating with a mean dry-film thickness of 120 µm, so the test time in the salt-spray test chamber was defined accordingly. Coatings of such characteristics and dry-film thickness are expected to have corrosion resistance in the C3 category, according to the coating manufacturer’s instructions, based on its characteristics and chemical composition. The duration of testing in the salt-spray test chamber for these characteristics in the coatings was 240 h, and was defined according to the norm HRN EN ISO 9227. Since the coating thickness is one of the input variables in this experiment, the values that were below and above the mean value in the same amount, then the remaining two time intervals for keeping specimens in the salt-spray test chamber, were defined for shorter and longer durations than the mentioned 240 h. Therefore, the time intervals for testing in the salt-spray test chamber were set to 120, 240, and 480 h. Before placing the specimens in the salt-spray test chamber, each specimen was subjected to a specific conditioning time after the coating was applied. In addition, a notch was incised in each specimen before placing them in the salt-spray test chamber. These specimens are presented in Figure 4. Figure 5 shows the test specimens inside the salt chamber workspace during the test, while Figure 6 shows the specimens after exposure to the salt-spray test chamber for a defined time interval.

### Testing of the Notch Corrosion

**Figure 7:**
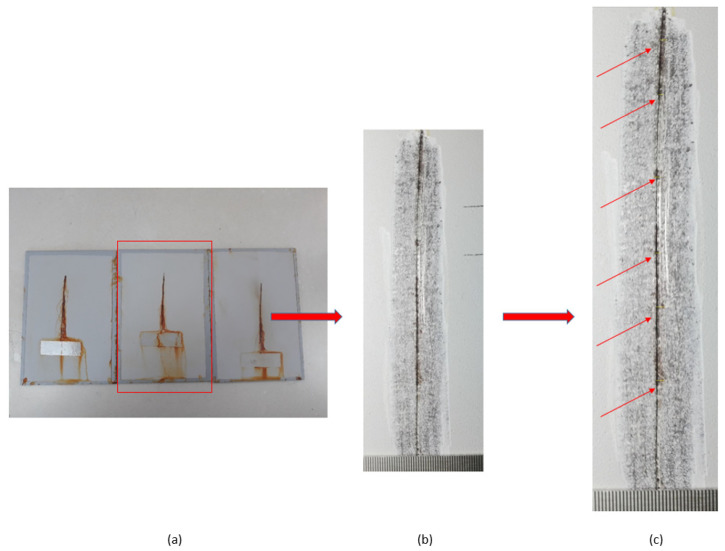
Notch corrosion test procedure: (**a**) the test sample after removal from the salt chamber; (**b**) removal of the coating around the notch; and (**c**) detection of places where the development of corrosion mechanisms occurred.

Testing of the notch corrosion was performed in accordance with the HRN EN ISO 4628-8 standard, and refers to the examination of protective properties. The test involves the assessment of coating deterioration, i.e., it determines the coating’s resistance to corrosion in certain service conditions. The test is carried out by removing the coating around the notch on the specimen after taking the specimens out of the salt-spray test chamber. There areas in which corrosion developed are detected, and the size of the corrosion product spread is measured at six points on the notch (Figure 7).

**Figure 8:**
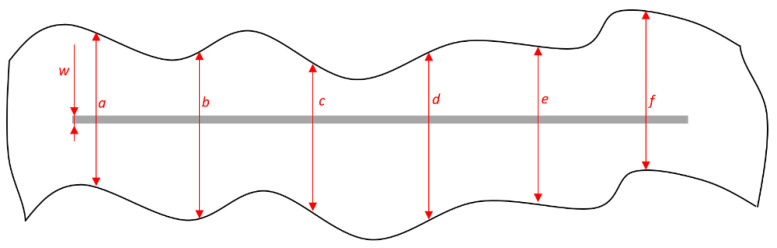
Measuring the size of corrosion product spread on specimen.

Figure 8 presents the points of measurement. When performing the measurement, it is necessary to consider the mutual distance between the corrosion mechanisms focal points to be measured, which should be at least 6 mm. The obtained data are entered into Formulas (1) and (2) in order to obtain a value that estimates the coating deterioration, i.e., that characterizes the protective effect of the coating [43]:

(1) $$d_{1}=\frac{a+b+c+d+e+f}{6}$$

(2) $$d=\frac{d_{1}-w}{2}$$

where the values *a*, *b*, *c*, *d*, *e,* and *f* refer to the measured values of the corrosion product spread in the area around the notch being affected by corrosion mechanisms; *d*_1_ refers to the mean value of measured sizes of corrosion spread around the notch; *w* refers to the width of initial notch; and *d* marks the value of notch corrosion.

## 3. Research Results

The results of the experimental runs, performed according to the central composite design (Table 2), are presented as the arithmetic mean of the response. The standard order run is applied to mark the specimens, according to the conventional method for the central composite design. Design Expert software was used for statistical analysis, as well as for random selection of the experiment run. Table 2 also provides an overview of the research results obtained after all three time intervals of keeping the specimens in the aggressive atmosphere of the salt-spray test chamber. The arithmetic mean was calculated for three repeated measurements. The principle of randomization was followed, and 117 samples (three for each experimental condition) were processed by the generated random experimental order.

### 3.1. Results Referring to Notch Corrosion Measurement after Exposure of Specimens to the Salt-Spray Test Chamber Atmosphere for a duration of 120 h

The analysis of the obtained data (as presented in Table 2) shows that the minimum response value was 0.01 mm, and the maximum response value was 0.26 mm. The arithmetic mean of the response was 0.1354 mm, while the standard deviation, i.e., the average deviation of each data from the arithmetic mean, was 0.0639 mm. When comparing the values of the linear model, the two-factor interaction (2FI) model, the quadratic model, and the cubic model, the quadratic model was the best for notch corrosion values according to different indicators in the experiment. The indicators used for selection of the model are shown in Table 4. The stated models were tested in relation to the *p* value for a specific model, the *p* value for the lack of fit, and the coefficients of determination.

Table 5 below shows the report obtained from Design Expert, referring to the analysis of variance of the proposed and selected quadratic model; it presents the dependence of notch corrosion on the input variables.

The coefficient of determination *R*^2^ refers to the share of explained variability (deviation of regression *y* from the arithmetic mean) in total variability (deviation of real *y* from the arithmetic mean), which is 0.8265. The adjusted coefficient of determination *R*^2^_adj_ is adjusted to the number of model members in relation to the number of runs, which is 0.7558. The predicted coefficient of determination *R*^2^_pred_ is 0.5748. Expression (3) shows regression model for the dependence of notch corrosion on the input variables (Factor *A*—anticorrosive pigment content, Factor *B*—conditioning time, Factor *C*—dry-film thickness). The values of the mentioned variables are coded for high factor levels as +1, and for low factor levels as –1, as stated in Table 1. Expression (4) refers to a regression model with real factor values where Factor *C* (dry-film thickness) equals the *D*1 value. Expression (5) refers to a regression model with real factor values where Factor *C* equals the *D*2 value, and Expression (6) refers to a regression model with real factor values where Factor *C* is equal to the *D*3 value.

*Notch corrosion* = 0.1733 − 0.0559 ∙ *A* + 0.0116 ∙ *B* + 0.0054 ∙ *C*1 + 0.0115 ∙ *C*2 + 0.0067 ∙ *AB* + 0.0082 ∙ *AC*1 + 0.0053 ∙ *AC*2 + 0.0180 ∙ *BC*1 − 0.0084 ∙ *BC*2 − 0.0242 ∙ *A*^2^ − 0.0375 ∙ *B*^2^ (3)

*Notch corrosion* = 0.046744 + 0.003267 ∙ *Anticorrosive pigment content* + 0.001572 ∙ *Conditioning time* + 0.000017 ∙ *Anticorrosive pigment content* ∙ *Conditioning time* − 0.001510 ∙ *Anticorrosive pigment content*^2^ − 4.0690 ∙ 10^–6^ ∙ *Conditioning time*^2^ (4)

*Notch corrosion* = 0.103485 + 0.002544 ∙ *Anticorrosive pigment content* + 0.001297 ∙ *Conditioning time* + 0.000017 ∙ *Anticorrosive pigment content* ∙ *Conditioning time* − 0.001510 ∙ *Anticorrosive pigment content*^2^ − 4.0690 ∙ 10^–6^ ∙ *Conditioning time*^2^ (5)

*Notch corrosion* = 0.105336 − 0.002144 ∙ *Anticorrosive pigment content* + 0.001284 ∙ *Conditioning time* + 0.000017 ∙ *Anticorrosive pigment content* ∙ *Conditioning time* − 0.001510 ∙ *Anticorrosive pigment content*^2^ − 4.0690 ∙ 10^–6^ ∙ *Conditioning time*^2^ (6)

### 3.2. Results Referring to Notch Corrosion Measurement after Exposure of Specimens to the Salt-Spray Test Chamber Atmosphere for a duration of 240 h

By analyzing the obtained data (as presented in Table 2), a minimum response value of 0.07 mm and a maximum response value of 0.29 mm were determined. The arithmetic mean of the response was 0.1797 mm, while the standard deviation, i.e., the average deviation of each datum from the arithmetic mean, was 0.0537 mm. When comparing values of the linear model, the two-factor interaction (2FI) model, the quadratic model and the cubic model, the linear model proved to be the best for notch corrosion values according to different indicators in the experiment. The indicators used for selection of the model are shown in Table 6. The stated models were tested in relation to the *p* value for a specific model, the *p* value for the lack of fit, and the coefficients of determination.

Table 7 shows the report obtained from Design Expert, referring to the analysis of variance of the proposed and selected linear model, in which the dependence of notch corrosion on the input variables is presented.

The coefficient of determination *R*^2^ refers to the share of explained variability (deviation of regression *y* from the arithmetic mean) in total variability (deviation of real *y* from the arithmetic mean), which is 0.7563. The adjusted coefficient of determination *R*^2^_adj_ is adjusted to the number of model members in relation to the number of runs, and it is 0.7276. The predicted coefficient of determination *R*^2^_pred_ is 0.6726. Expression (7) describes a regression model of the dependence of notch corrosion on the input variables (Factor *A*—anticorrosive pigment content, Factor *B*—conditioning time, Factor *C*—dry-film thickness). The values of the mentioned variables are coded for high factor levels as +1, and for low factor levels as –1, according to Table 1. Expression (8) shows a regression model with real factor values where Factor *C* (dry-film thickness) equals the *D*1 value. Expression (9) describes a regression model with real factor values where Factor *C* equals the *D*2 value, and Expression (10) refers to a regression model with real factor values where Factor *C* is equal to the *D*3 value.

*Notch corrosion* = 0.1797 − 0.0536 ∙ *A* − 0.0198 ∙ *B* + 0.0141 ∙ *C*1 − 0.0025 ∙ *C*2 (7)

*Notch corrosion* = 0.308997 − 0.013407 ∙ *Anticorrosive pigment content* − 0.000207 ∙ *Conditioning time* (8)

*Notch corrosion* = 0.292074 − 0.013407 ∙ *Anticorrosive pigment content* − 0.000207 ∙ *Conditioning time* (9)

*Notch corrosion* = 0.283613 − 0.013407 ∙ *Anticorrosive pigment content* − 0.000207 ∙ *Conditioning time* (10)

### 3.3. Results Referring to Notch Corrosion Measurement after Exposure of Specimens to the Salt-Spray Test Chamber Atmosphere for a duration of 480 h

Upon analyzing the obtained data (Table 2), it was determined that the minimum response value was 0.17 mm, and the maximum response value was 1.28 mm. The arithmetic mean of the response was 0.7549 mm, while the standard deviation was 0.2497 mm. When comparing values of the linear model, the two-factor interaction (2FI) model, the quadratic model and the cubic model, the linear model was the best for notch corrosion values according to the performed experiment. The indicators used for the selection of the model are shown in Table 8. The stated models were tested in relation to the *p* value for a specific model, the *p* value for the lack of fit, and the coefficients of determination.

Table 9 presents the report from Design Expert, referring to the analysis of variance of the proposed and selected linear model; it presents the dependence of notch corrosion on the input variables.

The coefficient of determination *R*^2^ refers to the share of explained variability (deviation of regression *y* from the arithmetic mean) in total variability (deviation of real *y* from the arithmetic mean), which is 0.6997. The adjusted coefficient of determination *R*^2^_adj_ is adjusted to the number of model members in relation to the number of runs, which is 0.6644. The predicted coefficient of determination *R*^2^_pred_ is 0.6016. Expression (11) shows a regression model of the dependence of notch corrosion on the input variables (Factor *A*—anticorrosive pigment content, Factor *B*—conditioning time, Factor *C*—dry-film thickness). The values of the mentioned variables are coded for high factor levels as +1, and for low factor levels as –1, according to Table 1. Expression (12) refers to a regression model with real factor values where Factor *C* (dry-film thickness) equals the *D*1 value. Expression (13) refers to a regression model with real factor values where Factor *C* equals the *D*2 value, and Expression (14) refers to a regression model with real factor values where Factor *C* is equal to the *D*3 value.

*Notch corrosion* = 0.7549 − 0.2202 ∙ *A* + 0.0233 ∙ *B* + 0.1505 ∙ *C*1 − 0.0364 ∙ *C*2 (11)

*Notch corrosion* = 1.19482 − 0.055039 ∙ *Anticorrosive pigment content* + 0.000243 ∙ *Conditioning time* (12)

*Notch corrosion* = 1.00789 − 0.055039 ∙ *Anticorrosive pigment content* + 0.000243 ∙ *Conditioning time* (13)

*Notch corrosion* = 0.930201 − 0.055039 ∙ *Anticorrosive pigment content* + 0.000243 ∙ *Conditioning time* (14)

**Figure 9:**
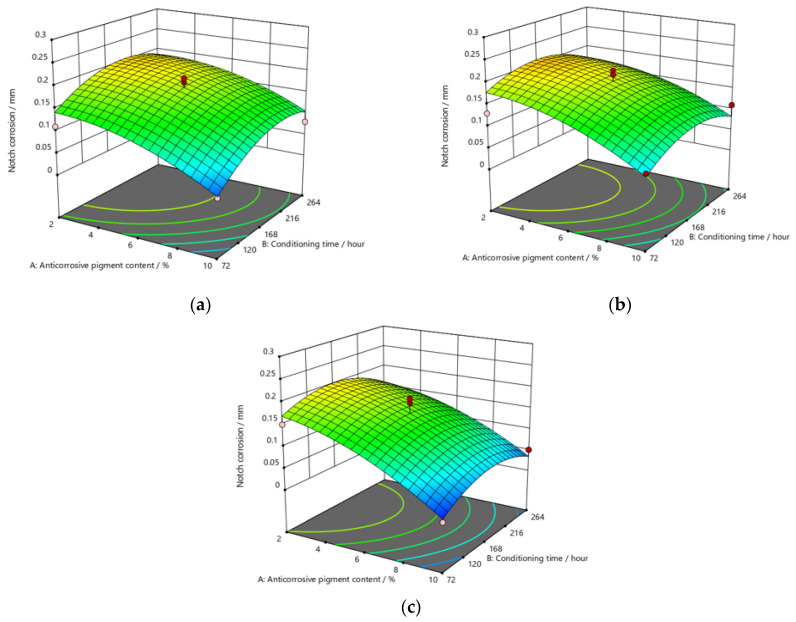
Response surface for regression model of notch corrosion for coating thickness of (**a**) *D*1, (**b**) *D*2, and (**c**) *D*3 values after 120 h in the salt-spray test chamber.

**Figure 10:**
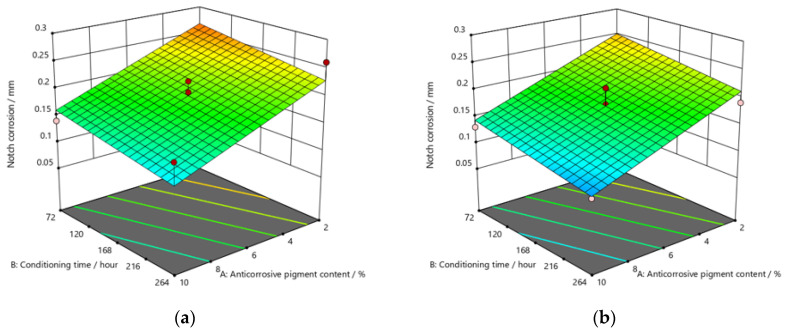

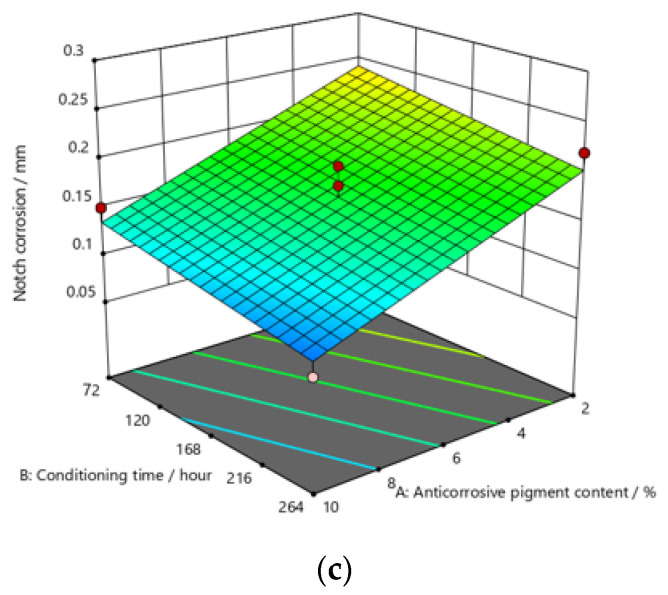
Response surface for regression model of notch corrosion for coating thickness of (**a**) *D*1, (**b**) *D*2, and (**c**) *D*3 values after 240 h in the salt-spray test chamber.

**Figure 11:**
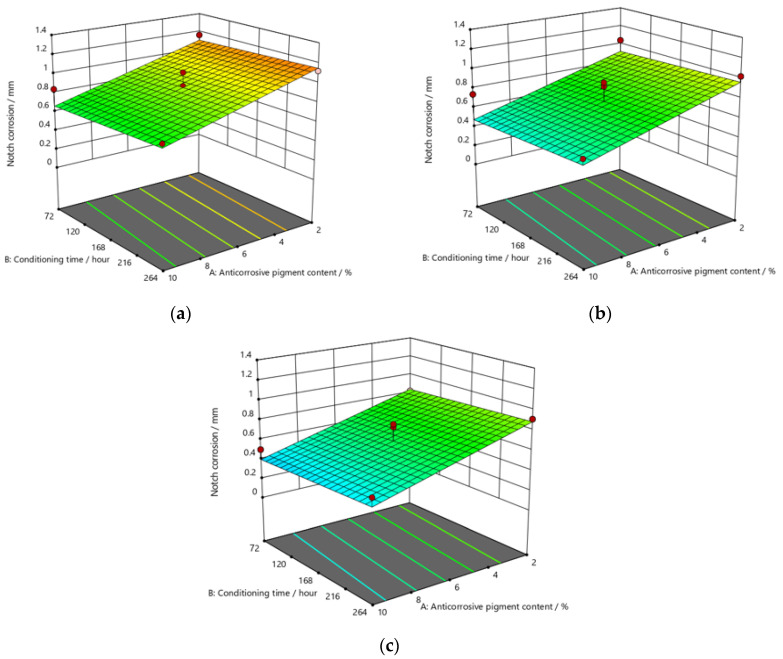
Response surface for regression model of notch corrosion for coating thickness of (**a**) *D*1, (**b**) *D*2, and (**c**) *D*3 values after 480 h in the salt-spray test chamber.

Figure 9, Figure 10 and Figure 11 refer to the graphic presentations of the models obtained by conducting experimental research.

## 4. Discussion and Analysis of Research Results

After exposing the specimens to the aggressive atmosphere of the salt-spray test chamber for a duration of 120 h, there were lower values of notch corrosion measured for coatings with a higher content of anticorrosive pigment, at all values of the conditioning time. Notch corrosion was additionally reduced with an increase in the dry-film thickness. It was clear that the notch corrosion values obtained in aggressive experimental conditions were not particularly high, i.e., they were within acceptable limits, which confirms the possibility of adding less anticorrosive pigment to the coating. This would lower production costs due to the reduced content of this component, without compromising the satisfactory protective properties of the product. The tests performed on the specimens after exposing them to the aggressive atmosphere of the salt-spray test chamber for 240 h led to the following conclusions: the measurement of notch corrosion showed that the lowest values were obtained with a higher content of anticorrosive pigment in the coating at a slightly longer conditioning time. Fewer corrosion mechanisms also occurred with an increase in the dry-film thickness in the applied coating. Detailed analysis proved that the corrosion values for the same content of anticorrosive pigment were not significantly different. This is interesting for practical reasons, because the application of a coating in service conditions usually allows a short conditioning time, since the coating is immediately exposed to aggressive reactants from the environment. However, even under such conditions, corrosion resistance will not be drastically reduced. After exposing specimens to the aggressive atmosphere of the salt-spray test chamber for 480 h, the experiment resulted in the following: the notch corrosion values were lower at a higher content of anticorrosive pigment for all values of the conditioning time. Moreover, the notch corrosion was lowered with an increase in the dry-film thickness of the applied coating. Unlike the previous two durations of specimens’ exposure to the aggressive atmosphere of the salt-spray test chamber that lasted for 120 and 240 h, in this case, the measured values of some samples were slightly higher than acceptable. Therefore, for the dry-film thickness of the *D*1 value, it is necessary to carefully determine the level to which the anticorrosive pigment content can be reduced, to retain the required corrosion resistance quality within acceptable values. In *D*2 and *D*3 coatings, the increase in the dry-film thickness can compensate for the decrease in the anticorrosive pigment content and prevent the development of corrosion mechanisms, which will result in lower notch-corrosion values. The notch corrosion values measured on specimens that were exposed to the aggressive atmosphere of the salt-spray test chamber for 120 and 240 h were still kept within the required limits. Therefore, lowering of the anticorrosive pigment content in the coating is justified because it can reduce production costs. The exposure of the specimens to the aggressive atmosphere of the salt-spray test chamber for 480 h requires much greater attention, because at lower dry-film thickness values, notch corrosion occurred above the acceptable limits. In this case, the anticorrosive pigment content in the coating should be slightly higher. It is possible to reduce the anticorrosive pigment content in the coating by increasing the dry-film thickness of the coating, thus increasing the barrier between the material and the aggressive environment, so that corrosion mechanisms do not develop beyond acceptable limits.

## 5. Conclusions

The addition of a higher concentration of anticorrosive pigment (from 2 to 10%) proportionally resulted in the formation of protective phosphates, to a greater extent, on the surface of the protected material (notch corrosion decreased to a lower value). Such a trend can be clearly seen in Figure 9, Figure 10 and Figure 11. The values obtained by measuring the notch corrosion decreased with the increase in values referring to the dry-film thickness of the coating. Such a trend was confirmed for all the durations of exposure of the specimens to the aggressive atmosphere of the salt-spray test chamber (120, 240, and 480 h).

Observing the thickness of the dry film coating becomes even more interesting if observed in combination with the content of the anti-corrosion pigment. If, for example, samples that have been exposed to a salt chamber for 480 h are observed, for a dry-film thickness of D1 and an anti-corrosion pigment content of 10%, a notch corrosion of 0.6 mm can be observed. If we then look at the dry-film thickness of the D2 coating for the same duration of exposure as the test specimens in the salt chamber, it is noted that the corrosion on the notch at a value of 0.6 mm occurred with an anticorrosive pigment content of approximately 0.8%, or with a dry-film thickness of D3 with an anti-corrosion pigment content of 0.6%.

Referring to the values of notch corrosion measured with regard to dependence on the conditioning time, it was confirmed that conditioning time affected specimens that were kept in the salt-spray test chamber for 240 h. In this research, lower values of notch corrosion that indicate better protective properties of the coating were measured in specimens with slightly higher values of conditioning time.

The mathematical models obtained from testing and statistical data processing come to the fore because they could be used for each precisely defined case of aggressive environment, to find the ratios of the previously described parameters that will give the best required properties, i.e., better resistance to corrosion. The optimization of the obtained parameters should not lead to higher-than-necessary dry-film thickness or concentrations of anti-corrosion pigment, for example, in the application of coatings, because this would not only exceed the required properties, but also significantly affect production costs. In contrast, the minimum values of the combinations of input parameters, below which the coating would not meet the required properties—and as such, would not be acceptable—should be known. One example of applying optimization based only on a diagram of the results indicates that increased aggressiveness of environmental conditions—as proven with specimens kept in the salt-spray test chamber for 480 h and with reduced dry-film thickness of the coating—enabled greater corrosion damage than acceptable. Still, it is worth noting that this can be prevented by adding a slightly higher amount of anticorrosive pigment to the coating.

Depending on the exposure of the coated structures to the aggressive environment, this research proves that it is possible to adjust the values of parameters to achieve the best protective properties of coatings, and to reduce the financial burden of their production by varying the combinations of certain coating parameters that respond well to aggressive conditions, such as those presented in this paper.

## Figures and Tables

**Table 1:** Coded values of the experiment design.

Coded Values	Factor 1—Anticorrosive Pigment Content (%)	Factor 2—Conditioning Time (Hours)	Factor 3—Coating Thickness		
−1.414	0.34	32	D1 (75–85 µm)	D2 (115–125 µm)	D3 (155–165 µm)
−1	2	72			
0	6	168			
1	10	264			
1.414	11.66	304			

**Table 2:** Central composite design with overview of obtained research results.

Factor 1—Anticorrosive Pigment Content (%)	Factor 2—Conditioning Time (Hours)	Factor 3—Coating Thickness	Notch Corrosion after 120 h in the Salt-Spray Test Chamber (mm)	Notch Corrosion after 240 h in the Salt-Spray Test Chamber (mm)	Notch Corrosion after 480 h in the Salt-Spray Test Chamber (mm)
2	72	*D*1 (75–85)	0.11	0.23	1.16
10	72	*D*1 (75–85)	0.03	0.14	0.84
2	264	*D*1 (75–85)	0.17	0.26	1.12
10	264	*D*1 (75–85)	0.08	0.16	0.75
0.34	168	*D*1 (75–85)	0.21	0.29	1.28
11.66	168	*D*1 (75–85)	0.06	0.08	0.3
6	32	*D*1 (75–85)	0.09	0.23	0.91
6	304	*D*1 (75–85)	0.18	018	1.08
6	168	*D*1 (75–85)	0.15	0.18	0.73
6	168	*D*1 (75–85)	0.16	0.22	0.75
6	168	*D*1 (75–85)	0.19	0.2	1.06
6	168	*D*1 (75–85)	0.2	0.19	0.87
6	168	*D*1 (75–85)	0.2	0.16	0.92
2	72	*D*2 (115–125)	0.13	0.21	1.04
10	72	*D*2 (115–125)	0.07	0.13	0.74
2	264	*D*2 (115–125)	0.13	0.19	1.02
10	264	*D*2 (115–125)	0.11	0.1	0.58
0.34	168	*D*2 (115–125)	0.26	0.2	0.74
11.66	168	*D*2 (115–125)	0.03	0.11	0.17
6	32	*D*2 (115–125)	0.14	0.25	0.53
6	304	*D*2 (115–125)	0.13	0.15	0.84
6	168	*D*2 (115–125)	0.14	0.15	0.87
6	168	*D*2 (115–125)	0.21	0.18	0.58
6	168	*D*2 (115–125)	0.2	0.21	0.91
6	168	*D*2 (115–125)	0.2	0.21	0.61
6	168	*D*2 (115–125)	0.16	0.21	0.71
2	72	*D*3 (155–165)	0.15	0.23	0.82
10	72	*D*3 (155–165)	0.01	0.15	0.5
2	264	*D*3 (155–165)	0.14	0.22	0.91
10	264	*D*3 (155–165)	0.05	0.08	0.53
0.34	168	*D*3 (155–165)	0.25	0.27	0.92
11.66	168	*D*3 (155–165)	0.02	0.07	0.24
6	32	*D*3 (155–165)	0.07	0.23	0.58
6	304	*D*3 (155–165)	0.06	0.1	0.63
6	168	*D*3 (155–165)	0.15	0.14	0.46
6	168	*D*3 (155–165)	0.18	0.16	0.78
6	168	*D*3 (155–165)	0.15	0.16	0.52
6	168	*D*3 (155–165)	0.12	0.2	0.63
6	168	*D*3 (155–165)	0.19	0.18	0.81

**Table 3:** Chemical composition of the base material [42].

Chemical Composition of Base Material/%			
C	P	S	N
0.17	0.05	0.05	≤0.007

**Table 4:** Simulation of four models in Design Expert for notch corrosion after 120 h in the salt-spray test chamber.

Model	*p* Value for the Model	*p* Value for the Lack of Fit	Adjusted Coefficient of Determination	Predicted Coefficient of Determination
Linear	<0.0001	0.0106	0.4895	0.4006
2FI	0.6991	0.0066	0.4578	0.1958
Quadratic	<0.0001	0.2083	0.7558	0.5748
Cubic	0.2691	0.2424	0.7802	0.2973

**Table 5:** Analysis of variance for the regression model for notch corrosion after 120 h in the salt-spray test chamber.

Source of Variance	Sum of Squares	Degrees of Freedom	Mean Squared Deviation	*F* Value	*p–*Value
Model	0.1281	11	0.0116	11.69	<0.0001
*A*—Anticorrosive pigment content	0.0751	1	0.0751	75.42	<0.0001
*B*—Conditioning time	0.0032	1	0.0032	3.26	0.0823
*C*—Coating thickness	0.0058	2	0.0029	2.93	0.0707
*AB*	0.0005	1	0.0005	0.5355	0.4706
*AC*	0.0022	2	0.0011	1.11	0.3445
*BC*	0.0039	2	0.0020	1.96	0.1600
*A*²	0.0122	1	0.0122	12.24	0.0016
*B*²	0.0293	1	0.0293	29.47	<0.0001
Residue	0.0269	27	0.0010		
Lack of fit	0.0179	15	0.0012	1.60	0.2083
Pure error	0.0090	12	0.0007		
Total	0.1550	38			

**Table 6:** Simulation of four models in Design Expert for notch corrosion after 240 h in the salt-spray test chamber.

Model	*p* Value for the Model	*p* Value for the Lack of Fit	Adjusted Coefficient of Determination	Predicted Coefficient of Determination
Linear	<0.0001	0.2192	0.7276	0.6726
2FI	0.0657	0.3696	0.7728	0.6628
Quadratic	0.3257	0.3755	0.7754	0.6429
Cubic	0.0910	0.7594	0.8298	0.6475

**Table 7:** Analysis of variance for the regression model for notch corrosion after 240 h in the salt-spray test chamber.

Source of Variance	Sum of Squares	Degrees of Freedom	Mean Squared Deviation	*F* Value	*p–*Value
Model	0.0828	4	0.0207	26.38	<0.0001
*A*—Anticorrosive pigment content	0.0690	1	0.0690	87.94	<0.0001
*B*—Conditioning time	0.0094	1	0.0094	12.03	0.0014
*C*—Coating thickness	0.0043	2	0.0022	2.77	0.0770
Residual	0.0267	34	0.0008		
Lack of fit	0.0197	22	0.0009	1.55	0.2192
Pure error	0.0070	12	0.0006		
Total	0.1095	38			

**Table 8:** Simulation of four models in Design Expert for notch corrosion after 480 h in the salt-spray test chamber.

Model	*p* Value for the Model	*p* Value for the Lack of Fit	Adjusted Coefficient of Determination	Predicted Coefficient of Determination
Linear	<0.0001	0.5483	0.6644	0.6016
2FI	0.9511	0.3949	0.6209	0.4331
Quadratic	0.2239	0.4346	0.6355	0.4097
Cubic	0.7818	0.2338	0.5837	–0.3407

**Table 9:** Analysis of variance for the regression model for notch corrosion after 480 h in the salt-spray test chamber.

Source of Variance	Sum of Squares	Degrees of Freedom	Mean Squared Deviation	*F* Value	*p–*Value
Model	1.66	4	0.4143	19.80	<0.0001
*A*—Anticorrosive pigment content	1.16	1	1.16	55.60	<0.0001
*B*—Conditioning time	0.0130	1	0.0130	0.6235	0.4352
*C*—Coating thickness	0.4810	2	0.2405	11.50	0.0002
Residue	0.7113	34	0.0209		
Lack of fit	0.4543	22	0.0206	0.9640	0.5483
Pure error	0.2570	12	0.0214		
Total	2.37	38			

## Data Availability

The data presented in this study are available from the corresponding author, upon reasonable request.
